# Racial and Ethnic Differences in Social Determinants of Health and Health-Related Social Needs Among Adults — Behavioral Risk Factor Surveillance System, United States, 2022

**DOI:** 10.15585/mmwr.mm7309a3

**Published:** 2024-03-07

**Authors:** Machell Town, Paul Eke, Guixiang Zhao, Craig W. Thomas, Jason Hsia, Carol Pierannunzi, Karen Hacker

**Affiliations:** ^1^Division of Population Health, National Center for Chronic Disease Prevention and Health Promotion, CDC; ^2^eLittle Communications Group, St. Louis, Missouri; ^3^National Center for Chronic Disease Prevention and Health Promotion, CDC.

**Summary**

**What is already known about this topic?**

Social determinants of health are the nonmedical factors that influence health outcomes.

**What is added by this report?**

Social isolation or loneliness and lack of social and emotional support were the most commonly reported measures among U.S. adults. The majority of prevalence estimates for adverse social determinants of health and health-related social needs were significantly higher across all other racial and ethnic groups except non-Hispanic Asian adults when compared with non-Hispanic White adults.

**What are the implications for public health practice?**

Decision makers and policymakers can use this information to understand and assess the impact of social determinants of health and health-related social needs on health and to evaluate interventions.

## Abstract

Social determinants of health (SDOH) are a broad array of social and contextual conditions where persons are born, live, learn, work, play, worship, and age that influence their physical and mental wellbeing and quality of life. Using 2022 Behavioral Risk Factor Surveillance System data, this study assessed measures of adverse SDOH and health-related social needs (HRSN) among U.S. adult populations. Measures included life satisfaction, social and emotional support, social isolation or loneliness, employment stability, food stability/security, housing stability/security, utility stability/security, transportation access, mental well-being, and health care access. Prevalence ratios were adjusted for age, sex, education, marital status, income, and self-rated health. Social isolation or loneliness (31.9%) and lack of social and emotional support (24.8%) were the most commonly reported measures, both of which were more prevalent among non-Hispanic (NH) American Indian or Alaska Native, NH Black or African American, NH Native Hawaiian or other Pacific Islander, NH multiracial, and Hispanic or Latino adults than among NH White adults. The majority of prevalence estimates for other adverse SDOH and HRSN were also higher across all other racial and ethnic groups (except for NH Asian) compared with NH White adults. SDOH and HRSN data can be used to monitor needed social and health resources in the U.S. population and help evaluate population-scale interventions.

## Introduction

Social determinants of health (SDOH) are the nonmedical factors that influence health outcomes. They are the conditions in which persons are born, live, learn, work, play, worship, and age that affect a wide range of health risks, functioning, and quality of life.* Examples of SDOH measures include economic stability, transportation availability, housing and food security, access to health care, built environment, and social connectedness (*1*). SDOH are driven by intersecting systematic influences such as economic policies and institutional racism that unequally affect different populations. SDOH and health-related social needs (HRSN) play a significant role in health status, health care utilization, and well-being of individual persons and populations (*2*). Whereas HRSN focus primarily on screening and connecting persons to resources and services to fulfill unmet social needs, SDOH exist at the community or population level and reflect the policies and environments that support health or create barriers to health (*2*). Some adverse SDOH have been linked to a higher risk for poor health outcomes, including chronic diseases (*3*,*4*).

This study measured the prevalence of adverse SDOH and HRSN across U.S. adult populations using data from the 2022 Behavioral Risk Factor Surveillance System (BRFSS). Understanding disparities in SDOH and HRSN among populations is essential to determining and deploying strategies toward advancing health equity. For the first time, data from a new Social Determinants and Health Equity (SD/HE) module in BRFSS were used to investigate adverse SDOH and HRSN by race and ethnicity in the United States.

## Methods

### Data Source

BRFSS is a state-based landline and cellular telephone survey of noninstitutionalized U.S. civilian residents aged ≥18 years.^†^ BRFSS collects data on health-related risk behaviors, chronic diseases and conditions, health care access, and use of preventive services in all 50 states, the District of Columbia, and participating U.S. territories. The optional SD/HE module was introduced in 2022. Details of the 2022 BRFSS survey and SD/HE module are described elsewhere (*5*); data were collected by 39 states, District of Columbia, Puerto Rico, and U.S. Virgin Islands.^§^ SD/HE module questions were developed based on the Center for Medicare & Medicaid Services’ Accountable Health Communities Health-Related Social Needs Screening Tool^¶^ and from a previous BRFSS SDOH optional module administered in 2017.** SDOH measures include employment instability, food insecurity, housing insecurity, utility insecurity, and lack of reliable transportation. HRSN measures included life dissatisfaction, lack of social and emotional support, social isolation or loneliness, receiving food stamps or Supplemental Nutrition Assistance Program (SNAP), and mental stress. Two additional adverse SDOH measures, lack of health insurance and cost barrier for needed medical care, were from the BRFSS core section (Box).

BOX **Adverse social determinants of health and health-related social needs measures — Behavioral Risk Factor Surveillance System, United States, 2022**

**Life dissatisfaction**

- Defined with a response of “dissatisfied/very dissatisfied” to the question, “In general, how satisfied are you with your life? Are you…”

**Lack of social and emotional support**

- Defined with a response of “sometimes/rarely/never” to the question, “How often do you get the social and emotional support that you need? Is that…”

**Social isolation or loneliness**

- Defined with a response of “always/usually/sometimes” to the question, “How often do you feel socially isolated from others? Is it…”

**Loss or reduced hours of employment**

- Defined with a response of “yes” to the question, “In the past 12 months, have you lost employment or had hours reduced?”

**Receiving food stamps or SNAP**

- Defined with a response of “yes” to the question, “During the past 12 months, have you received food stamps, also called SNAP, the Supplemental Nutrition Assistance Program on an EBT card?”

**Food insecurity**

- Defined with a response of “always/usually/sometimes” to the question, “During the past 12 months, how often did the food that you bought not last, and you didn’t have money to get more? Was that…”

**Housing insecurity**

- Defined with a response of “yes” to the question, “During the last 12 months, was there a time when you were not able to pay your mortgage, rent, or utility bills?”

**Experiencing threat to shut off utility services**

- Defined with a response of “yes” to the question, “During the last 12 months, was there a time when an electric, gas, oil, or water company threatened to shut off services?”

**Lack of reliable transportation**

- Defined with a response of “yes” to the question, “During the past 12 months, has a lack of reliable transportation kept you from medical appointments, meetings, work, or from getting things needed for daily living?”

**Mental stress**

- Defined with a response of “always/usually” to the question, “Stress means a situation in which a person feels tense, restless, nervous or anxious, or is unable to sleep at night because their mind is troubled all the time. Within the last 30 days, how often have you felt this kind of stress? Was it…”

**Lack of health insurance**

- Defined with a response of “no coverage of any type” to the question, “What is the current primary source of your health insurance?”

**Cost barrier for needed medical care**

- Defined with a response of “yes” to the question, “Was there a time in the past 12 months when you needed to see a doctor but could not because you could not afford it?”

**Abbreviations:** EBT = electronic benefits transfer; SNAP = Supplemental Nutrition Assistance Program.

Prevalence of adverse SDOH and HRSN were examined by race and ethnicity, which were categorized as non-Hispanic (NH) American Indian or Alaska Native (AI/AN), NH Asian (Asian), NH Black or African American (Black), NH Native Hawaiian or other Pacific Islander (NH/OPI), NH White (White), NH multiracial (multiracial), or Hispanic or Latino (Hispanic) based on self-identified race and ethnicity information. The analysis included 323,877 participants (among 338,778 survey respondents) with complete demographic and general health status information.

### Data Analysis

Those who responded “don’t know/not sure,” refused to answer, or had missing responses for demographic variables (except for those with unknown income) were excluded. Participants with missing information for a specific SDOH or HRSN were excluded from the respective analyses.

Weighted^††^ prevalence estimates were calculated overall and by racial and ethnic group, U.S. Census Bureau regions, and covariates (age, sex, education, marital status, income, and self-rated health). Statistical significance was determined based on whether there was an overlap between 95% CIs for any two estimates. Adjusted prevalence estimates were obtained by conducting log-linear regression analyses with a robust variance estimator, which adjusted for covariates. Analyses were conducted using SAS-callable SUDAAN (version 11.0.3; RTI International) to account for the complex survey design. This activity was reviewed by CDC, deemed not research, and was conducted consistent with applicable federal law and CDC policy.^§§^

## Results

The most commonly reported adverse SDOH or HRSN were social isolation or loneliness (31.9%) and lack of social and emotional support (24.8%), which are proxies for social connectedness (Supplementary Table, https://stacks.cdc.gov/view/cdc/148477). Receiving food stamps or SNAP was most prevalent among Black adults (21.9%) and AI/AN adults (21.3%); lack of reliable transportation was most prevalent among AI/AN adults (16.2%). The following were most prevalent among NH/OPI adults: lack of social and emotional support (38.3%), loss or reduced hours of employment (21.4%), food insecurity (29.0%), housing insecurity (22.8%), and experiencing threat to shut off utility services (19.2%). Life dissatisfaction (11.2%) and social isolation or loneliness (41.0%) were most prevalent among multiracial adults. Lack of health insurance (21.0%) was most prevalent among Hispanic adults. The lowest prevalences of most adverse SDOH and HRSN measures were among Asian and White adults (Supplementary Table, https://stacks.cdc.gov/view/cdc/148477).

### Differences by Demographics and Health Status

The prevalence of adverse SDOH and HRSN also differed by other demographic characteristics and by general health status (Supplementary Table, https://stacks.cdc.gov/view/cdc/148477). For example, with increasing age, educational level, and household income, the prevalence of adverse SDOH and HRSN generally decreased. Adults who reported fair or poor self-rated health had the highest prevalence for all adverse SDOH and HRSN. Adults living in the U.S. Census Bureau South Region had the highest prevalences of receiving food stamps or SNAP, food insecurity, experiencing threat to shut off utility services, lack of health insurance, and cost barrier for needed medical care.

### Adjusted Analyses

After adjustment for covariates (Table), when compared with that of White adults, the prevalence of life dissatisfaction was 24% higher for multiracial adults, 14% lower for Black adults, and 33% lower for Hispanic adults; lack of social and emotional support ranged from 6% more prevalent in the Hispanic group to 76% more prevalent in the Asian group. Across all other racial and ethnic groups compared with White adults, the majority of prevalence estimates were higher for loss or reduced hours of employment (22% to 73%), receiving food stamps or SNAP (31% to 77%), food insecurity (35% to 133%), housing insecurity (34% to 105%), experiencing a threat to shut off utility services (50% to 149%, except for 39% lower among Asian adults), lack of reliable transportation (8% to 86%), and cost barrier for needed medical care (23% to 49%). Lack of health insurance coverage was 92% more prevalent for Hispanic adults than for White adults. The prevalence of mental stress was lower for three groups when compared with White adults: 22% less for Hispanic adults, 25% less for Black adults, and 39% less for Asian adults.

**TABLE:** Adjusted* prevalences and adjusted* prevalence ratios for having adverse social determinants of health and health-related social needs, by race and ethnicity among adults — Behavioral Risk Factor Surveillance System, United States, 2022

Characteristic	Race and ethnicity,^†^ no. (95% CI)								
	AI/AN	Asian	Black or African American	NH/OPI	White	Hispanic or Latino	Multiracial		
**Respondents, no.^§^**	4,750	7,549	25,851	690	245,585	33,451	6,001		
**Life dissatisfaction**									
AP	6.7 (5.5–8.2)	6.0 (4.8–7.4)	5.9 (5.4–6.5)	4.9 (2.6–9.2)	6.9 (6.7–7.2)	4.7 (4.2–5.1)		8.6 (7.5–9.9)	
APR	0.97 (0.79–1.19)	0.86 (0.69–1.07)	0.86 (0.78–0.94)	0.70 (0.37–1.33)	Ref	0.67 (0.61–0.75)		1.24 (1.08–1.43)	
**Lack of social and emotional support**									
AP	26.8 (24.3–29.6)	39.5 (36.7–42.5)	29.3 (28.3–30.4)	36.3 (30.3–43.4)	22.5 (22.1–22.9)	23.8 (22.9–24.8)		27.2 (25.2–29.3)	
APR	1.19 (1.08–1.32)	1.76 (1.63–1.89)	1.30 (1.25–1.36)	1.61 (1.35–1.93)	Ref	1.06 (1.01–1.11)		1.21 (1.12–1.31)	
**Social isolation or loneliness**									
AP	32.5 (29.7–35.4)	33.0 (30.6–35.6)	32.4 (31.2–33.6)	37.9 (31.9–44.9)	32.4 (32.0–32.8)	29.3 (28.4–30.3)		36.4 (34.2–38.8)	
APR	1.00 (0.92–1.09)	1.02 (0.94–1.10)	1.00 (0.96–1.04)	1.17 (0.98–1.39)	Ref	0.90 (0.87–0.94)		1.12 (1.05–1.20)	
**Loss or reduced hours of employment**									
AP	13.4 (11.3–15.8)	11.7 (9.9–13.7)	15.2 (14.3–16.2)	18.9 (14.1–25.4)	10.9 (10.6–11.2)	14.4 (13.7–15.1)		16.1 (14.4–18.1)	
APR	1.22 (1.03–1.45)	1.07 (0.91–1.26)	1.39 (1.30–1.49)	1.73 (1.29–2.33)	Ref	1.32 (1.24–1.39)		1.48 (1.31–1.66)	
**Receiving food stamps or SNAP**									
AP	15.2 (13.4–17.2)	10.6 (8.6–13.0)	17.7 (16.9–18.5)	11.0 (7.9–15.4)	10.0 (9.7–10.3)	13.1 (12.5–13.7)		15.1 (13.7–16.6)	
APR	1.52 (1.34–1.72)	1.05 (0.85–1.30)	1.77 (1.68–1.86)	1.10 (0.79–1.54)	Ref	1.31 (1.24–1.38)		1.50 (1.36–1.67)	
**Food insecurity**									
AP	18.4 (16.8–20.2)	13.0 (11.0–15.4)	20.0 (19.1–20.9)	26.2 (19.5–35.3)	11.2 (10.9–11.6)	15.2 (14.6–15.9)			15.8 (14.0–17.8)
APR	1.64 (1.49–1.80)	1.16 (0.97–1.38)	1.78 (1.68–1.87)	2.33 (1.73–3.15)	Ref	1.35 (1.28–1.43)			1.40 (1.24–1.59)
**Housing insecurity**									
AP	15.1 (13.3–17.1)	8.5 (6.9–10.5)	17.6 (16.8–18.5)	19.6 (14.3–26.9)	9.6 (9.3–9.9)	12.8 (12.2–13.4)			14.0 (12.4–15.8)
APR	1.58 (1.39–1.79)	0.89 (0.72–1.10)	1.84 (1.73–1.95)	2.05 (1.49–2.81)	Ref	1.34 (1.26–1.41)			1.46 (1.29–1.65)
**Experiencing threat to shut off utility services**									
AP	10.1 (8.8–11.7)	4.1 (2.9–5.6)	12.5 (11.7–13.3)	16.6 (11.7–23.3)	6.6 (6.4–6.9)	6.7 (6.2–7.1)			10.0 (8.6–11.5)
APR	1.53 (1.32–1.77)	0.61 (0.44–0.85)	1.88 (1.75–2.02)	2.49 (1.76–3.52)	Ref	1.00 (0.92–1.09)			1.50 (1.29–1.74)
**Lack of reliable transportation**									
AP	11.9 (10.5–13.5)	7.0 (5.6–8.7)	10.3 (9.6–11.0)	13.6 (8.4–22.0)	7.3 (7.0–7.6)	7.9 (7.4–8.4)			11.2 (9.8–12.8)
APR	1.64 (1.44–1.87)	0.96 (0.77–1.20)	1.41 (1.31–1.53)	1.86 (1.15–3.02)	Ref	1.08 (1.00–1.17)			1.54 (1.34–1.77)
**Mental stress**									
AP	15.6 (13.7–17.8)	9.6 (8.1–11.5)	12.0 (11.2–12.7)	18.7 (14.8–23.7)	15.9 (15.5–16.2)	12.3 (11.6–13.0)			16.8 (15.2–18.5)
APR	0.98 (0.86–1.12)	0.61 (0.51–0.73)	0.75 (0.71–0.81)	1.18 (0.93–1.50)	Ref	0.78 (0.73–0.83)			1.06 (0.96–1.17)
**Lack of health insurance**									
AP	7.5 (6.2–9.1)	6.2 (5.0–7.6)	7.1 (6.5–7.8)	8.5 (5.8–12.4)	6.7 (6.5–7.0)	12.9 (12.4–13.5)			7.3 (6.1–8.7)
APR	1.11 (0.91–1.36)	0.91 (0.74–1.13)	1.06 (0.96–1.17)	1.26 (0.86–1.84)	Ref	1.92 (1.81–2.04)			1.08 (0.90–1.30)
**Cost barrier for needed medical care**									
AP	11.4 (9.8–13.2)	8.6 (7.4–9.9)	11.2 (10.5–11.9)	15.6 (12.1–20.2)	10.5 (10.2–10.8)	12.9 (12.4–13.6)			13.7 (12.4–15.1)
APR	1.09 (0.93–1.26)	0.81 (0.70–0.95)	1.06 (0.99–1.13)	1.49 (1.15–1.92)	Ref	1.23 (1.17–1.30)			1.30 (1.18–1.44)

**Abbreviations: **AI/AN = American Indian or Alaska Native; AP = adjusted prevalence; APR = adjusted prevalence ratio; NH/OPI = Native Hawaiian or other Pacific Islander; Ref = referent group; SNAP = Supplemental Nutrition Assistance Program.

* Adjusted for age, sex, education, marital status, household income, and self-rated health.

^†^ Persons of Hispanic or Latino (Hispanic) origin might be of any race but are categorized as Hispanic; all racial groups are non-Hispanic.

**^§^** Because of some missing data, the number of respondents for individual social determinants of health and health-related social needs might be smaller than the number of total respondents.

## Discussion

In this large state-based survey of adverse SDOH and HRSN among U.S. adults, significant differences were reported among racial and ethnic groups in measures of social and emotional support, employment instability, food insecurity, housing insecurity, and utility and transportation instability. Estimates indicate elevated prevalences of adverse SDOH and HRSN among AI/AN, Black, NH/OPI, multiracial, and Hispanic adults when compared with White adults. Most adverse SDOH and HRSN estimates were not significantly different between Asian and White adults. Adults who reported having fair or poor health were more likely to have adverse SDOH and HRSN than those reporting better health. Disparities in chronic disease prevalence, severity, complications, and management, as well as related risk factors among racial and ethnic groups, are well documented (*6*). For example, racial and ethnic differences in cardiovascular disease mortality among U.S. adults that are not indicative of biologic differences but intersecting systematic influences are correlated with adverse SDOH (*7*,*8*).

This study identified the extent of differences in adverse SDOH and HRSN among racial and ethnic populations, and by U.S. Census Bureau regions, demographic characteristics, and general health status. Findings are consistent with the differential impact that societal structural and systemic infrastructure have on SDOH and HRSN among racial and ethnic populations in the United States (*9*). Further studies using the BRFSS SD/HE module will examine which SDOH and HRSN are most relevant to specific health outcomes and whether addressing these SDOH and HRSN could lead to improvement in health equity.

### Limitations

The findings in this report are subject to at least five limitations. First, the BRFSS SD/HE module was not administered in all jurisdictions, so the study sample is not representative of the entire U.S. adult population. Second, self-reported survey data are susceptible to recall bias and social desirability bias. Third, missing data on income and some of the SDOH measures might have introduced information bias. Fourth, the analysis did not stratify by other demographic variables that could mask disparities. Finally, this study did not consider the impact of other SDOH measures such as racism and built environment.

### Implications for Public Health Practice

This information has implications for developing more strategic and effective programs that address health disparities. For example, increased economic resources and social belonging interventions can improve health (*10*). Information on the differential prevalence of adverse SDOH and HRSN across demographic characteristics can be helpful in effective allocation of resources. The public health community, the social service system, policymakers, the health care system, and others can use this information to address the SDOH and HRSN that influence health. Trends in SDOH and HRSN measures can be monitored in the U.S. population and can help evaluate population-scale interventions.

## Footnotes

* https://health.gov/healthypeople/priority-areas/social-determinants-health

†
https://www.cdc.gov/brfss/

§ Alabama, Alaska, Arizona, California, Connecticut, Delaware, District of Columbia, Florida, Georgia, Idaho, Indiana, Iowa, Kansas, Kentucky, Maine, Maryland, Massachusetts, Michigan, Minnesota, Mississippi, Missouri, Montana, Nebraska, Nevada, New Hampshire, New Jersey, New Mexico, North Carolina, Ohio, Oklahoma, Rhode Island, South Carolina, Tennessee, Texas, Utah, Vermont, Washington, West Virginia, Wisconsin, Wyoming, Puerto Rico, and U.S. Virgin Islands.

¶
https://innovation.cms.gov/innovation-models/ahcm

**
https://www.cdc.gov/brfss/questionnaires/pdf-ques/2017_BRFSS_Pub_Ques_508_tagged.pdf

††
https://www.cdc.gov/brfss/annual_data/2022/pdf/2022-Weighting-Description-508.pdf

§§ 45 C.F.R. part 46.102(l)(2), 21 C.F.R. part 56; 42 U.S.C. Sect. 241(d); 5 U.S.C. Sect. 552a; 44 U.S.C. Sect. 3501 et seq.
